# Exploiting natural variation and genetic manipulation of stomatal conductance for crop improvement

**DOI:** 10.1016/j.pbi.2019.01.003

**Published:** 2019-06

**Authors:** Michele Faralli, Jack Matthews, Tracy Lawson

**Affiliations:** School of Biological Sciences, University of Essex, Wivenhoe Park, Colchester, CO4 3SQ, United Kingdom

## Abstract

Rising global temperatures and more frequent episodes of drought are expected to drive reductions in crop yield, therefore new avenues for improving crop productivity must be exploited. Stomatal conductance (*g_s_*) balances plant CO_2_ uptake and water loss, therefore, greatly impacting the cumulative rate of photosynthesis and water use over the growing season, which are key determinants of crop yield and productivity. Considerable natural variation exists in stomatal anatomy, biochemistry and behavioural characteristics that impact on the kinetics and magnitude of *g_s_* and thus gaseous exchange between the plant and atmosphere. Exploiting these differences in stomatal traits could provide novel breeding targets for new crop varieties that are potentially more water use efficient and have the ability to maintain and/or maximize yield in a range of diverse environments. Here we provide an overview of variation in stomatal traits and the impact these have on *g_s_* behaviour, as well as the potential to exploit such variation and genetic manipulation for crop improvement.

## Highlights

• Stomatal conductance is a major determinant of crop yield.
• The speed of stomatal response to changing environmental conditions greatly impacts photosynthesis and water use.
• Existing natural variation in the magnitude and rapidity of stomatal conductance is a potential target for future breeding.
• Genetic manipulation of stomatal conductance has the potential to improve crop performance.

**Current Opinion in Plant Biology** 2019, **49**:1–7

This review comes from a themed issue on **Physiology and metabolism**

Edited by **Elizabeth A Ainsworth** and **Elizabete Carmo-Silva**

For a complete overview see the Issue and the Editorial

Available online 7th March 2019

**https://doi.org/10.1016/j.pbi.2019.01.003**

1369-5266/© 2019 The Authors. Published by Elsevier Ltd. This is an open access article under the CC BY license (http://creativecommons.org/licenses/by/4.0/).

## Stomatal conductance influences crop photosynthesis and yield

Stomata govern gaseous diffusion between the leaf and the external atmosphere, regulating CO_2_ assimilation, water loss and evaporative cooling. Stomata continually adjust aperture in response to external environmental cues (e.g. light), plant water status [1], and internal signals, that may be hormonal (e.g. ABA) [2], circadian [3], and/or a currently unidentified ‘mesophyll signal’ [4,5], to maintain an appropriate balance between CO_2_ uptake and water loss. Over the long-term and under steady-state, non-limiting conditions, stomatal conductance (*g_s_*) has been reported to correlate strongly with the rate of photosynthesis (*A*) [6], with high *g_s_* generally associated with high *A* and yield [7]. However, short-term dynamic changes in the environment result in a lack of synchrony between *g_s_* and *A*, as stomatal responses to changing environmental cues are often substantially slower than those observed in *A*, resulting in a temporal disconnect between *A* and *g_s_* that can limit photosynthetic carbon assimilation and reduce plant water use efficiency (*W_i_*, carbon assimilation as a ratio of water lost) [5,8,9^••^]. Stomatal conductance is determined by both anatomical and behavioural characteristics, yet both vary greatly between and within species, as well as between [10] and within leaves [11], resulting in significant variation in stomatal behaviour and absolute *g_s_* [12].

## Anatomical characteristics determine the rate of *g_s_*

Anatomical features such as stomatal density (SD), size and maximum pore area, determine the calculated theoretical maximum stomatal conductance (*g_s_*_max_) [13], whilst the control of stomatal opening and closure determine ‘operational’ or measured *g_s_*, that is the fraction of *g_s_*_max_ at which the leaf operates [14]. A positive relationship between SD and *g_s_* has been reported within species [15], which often, but not always [16] translates into high *A* [17,18]. For example, [19] reported that increased SD in two near isogenic lines of barley did not result in increased *g_s_* due to a concurrent decrease in stomatal size. Stomatal density is also positively related to photosynthetic capacity, with several studies illustrating increases in operational and maximum *g_s_* with increases in photosynthetic potential [20,21]. Furthermore, it is well established that significant natural variation in photosynthetic capacity exists between [22] and within species [23^•^,24^•^]. Stomatal size and SD also vary greatly within and between plant species [10], with differences often driven by changes in the growth environment [25], including [CO_2_] [26], light intensity and spectral quality [27]. There are numerous studies that have also demonstrated significant variation in stomatal anatomical characteristics within species, cultivars, genotypes and ecotypes. For example, [28] examined 62 wild Arabidopsis accessions and reported significant variation in SD that was also related to other epidermal traits, including cell size, stomatal index and patterning, suggesting a common genetic basis. In [29] varietal differences in SD and aperture in rice genotypes were shown, which [16] demonstrated the importance of variation in stomatal length that resulted in genotypic variation in *g*_s_. Variation in SD has also been associated with differences in drought resistance, as well as photosynthetic rates in wheat cultivars [30]. Therefore, natural variation in stomatal characteristics represents an unexploited genetic resource for improving *g_s_*, *A* and plant performance. Although variation in SD is well-established there is limited information on the impact of stomatal behaviour and/or kinetics on *A*, *W_i_* and plant productivity.

## Variation in stomatal anatomy impacts on dynamic *g_s_* responses

**Figure 1:**
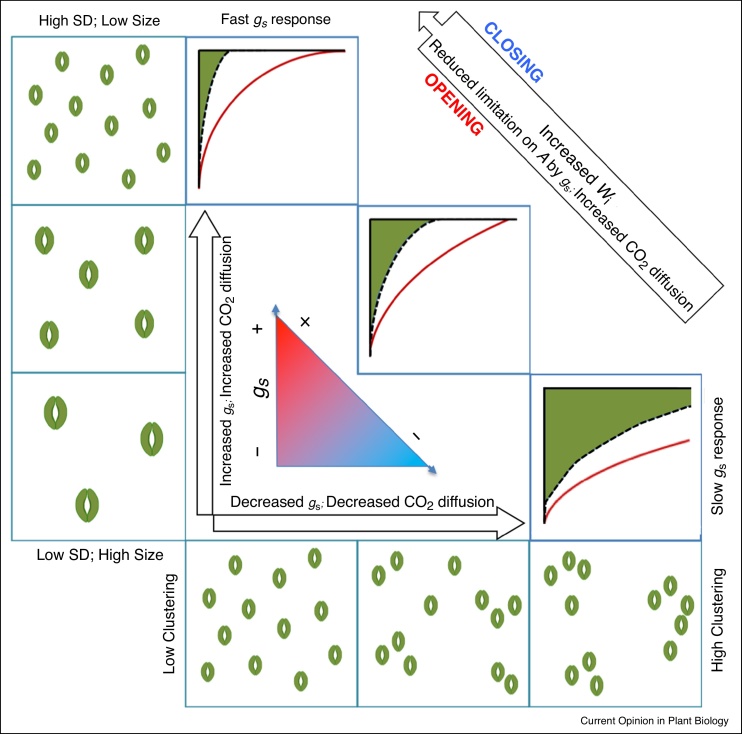
Diagram representing the influence of changes in stomatal anatomy (density and size; left panels, stomatal clustering; lower panels) on stomatal conductance (*g_s_*, arrows) and the rate of *g_s_* response (red lines). The impact of anatomical traits on carbon gain (*A,* dashed lines), the limitation of *A* by *g_s_* (green area) and water use efficiency (*W_i_*) are illustrated. The influence of stomatal density and size (vertical arrow) and stomatal clustering (horizontal arrow) on the rate of *g_s_* response and the maximum or operational value of *g_s_* is highlighted.

**Figure 2:**
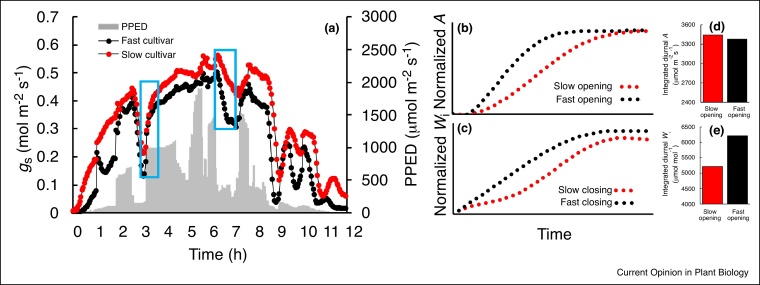
Diurnal time course of *g_s_* in two wheat cultivars with contrasting rapidity **(a)** under a dynamic light regime. Examples (blue sections) of the impact of slow and fast *g_s_* responses on *A* after a step increase in light **(b)**; and *W_i_* after a step decrease in light **(c)**. The integrated daily values of *A***(d)** and *W_i_***(e)** for cultivars with fast and slow stomatal responses is illustrated.

Modifications in SD have been reported to negatively correlate with stomatal size [25], which influences not only *g_s_* but also the speed at which stomata respond to changing environmental conditions [31,9^••^]. Several recent studies have demonstrated that stomatal kinetics are determined by anatomical attributes including stomatal morphology and shape [31,9^••^], size and density [32], patterning [33] and the presence or absence of subsidiary cells [9^••^,^34^], and that manipulation of these features could have positive effects on the efficiency of carbon assimilation and water use [35,36^•^]. Figure 1 shows the predicted impact of anatomical characters such as stomatal density and size on the magnitude and rapidity of the *g_s_* response to a step increase in light intensity, based on the literature [9^••^,^31^,^32^,^33^]. Leaves with a greater number of smaller stomata would be expected to have more rapid stomatal responses and a higher overall *g_s_* compared with leaves that had lower density and larger stomata. Additionally, stomatal patterning defects (i.e. stomatal clustering) have been reported to result in slower *g_s_* responses and lower *g_s_* values. [32] illustrated that the maximum rate of stomatal opening is driven by the surface-to-volume ratio of stomata, attributed to changes in SD and size, as species with higher stomatal densities and smaller stomata exhibited more rapid *g_s_* kinetics [31]. [9^••^] Quantified the impact of slow stomatal opening, in a range of species including crops, and determined on average a 10% limitation on carbon assimilation, which could equate to substantial losses in carbon gain over the course of the day, potentially negatively impacting productivity and yield [37,38]. In contrast, slow stomatal closure results in a significant decrease in intrinsic water use efficiency (*W_i_*) and resource use [9^••^,39^••^] thus potentially accellerating early soil water exhaustion [40]. Figure 2 highlights the impact on *A* of variation in the speed of stomatal opening and closure, between two wheat varieties (Figure 2a). Slow increases in *g_s_* limit CO_2_ diffusion, reducing *A* (Figure 2b + d); whilst slow decreases in *g_s_* result in lower *W_i_* (Figure 2c + e). Synchronized behaviour and close coupling of *A* and *g_s,_* therefore, have the potential to enhance carbon gain and *W_i_*, and in turn improve performance, productivity and yield [17,39^••^]. The wheat cultivars measured in Figure 2 showed little difference in *A* (Figure 2d) between the fast and slow *g_s_* responding cultivars, (most likely due to the greater *g_s_* in the slower responding cultivar), whilst *W_i_* (Figure 2e) was much greater in the cultivar with the faster *g_s_* responses.

Although substantial progress has been made in linking stomatal anatomy to function, the size and density of stomata are not the only determinants of the speed of response [9^••^], with stomatal patterning [33,41^•^] and guard cell biochemistry [17] also playing key roles. In fact, stomatal clustering has been shown to decrease *g_s_* and, therefore *A*, without any change in overall SD and size [33], and was attributed to reduced guard cell function and increased hydraulic competition with neighbouring guard cells [33,41^•^] (see Figure 1). Guard cell movement is the cumulative sum of net solute fluxes (e.g. K^+^, Cl^−^ and Malate) integrated over time and transported across the plasma membrane and the tonoplast [17,36^•^]. The density and the activity of the guard cell membrane transporters determine solute transport capacity and, inevitably, the speed and magnitude of stomatal movement [42]. Inter-specific variation in guard cells solute flux has been previously shown [17], corroborating the idea that stomatal movement is not only dependent on anatomical factors. Optimization of solute fluxes in guard cells has the potential to enhance stomatal rapidity and provides another unexploited target for crop breeding and should be given greater consideration in future research efforts.

## Genetic manipulation of *g_s_*

As *A* is strongly correlated with *g_s_* a greater emphasis should be placed on recognising *g_s_* as a major target to improve crop yields and optimize water use. There are multiple examples of the genetic manipulation of SD successfully altering *g_s_* and influencing plant performance. Work by Gray *et al.* produced mutants with altered stomatal density by manipulating epidermal patterning factor genes [43]. Overexpression of the epidermal patterning factor EPF2 has been shown to improve long-term *W*_*i*_ without adversely affecting photosynthetic capacity [44] whilst also improving drought tolerance [35]. This model has been successfully applied to improve drought tolerance in barley [45^•^]. In contrast, [46] manipulated another member of the EPF family, the mesophyll driven EPF9 (STOMAGEN), which increased SD and *g_s_* resulting in a 30% increase in *A*, although a 40% decrease in *W_i_* and no significant increase on growth was reported [47]. The above findings highlight that manipulation of stomatal anatomy could be a potential mechanism to increase *g_s_* and improve crop productivity and yield. However, it is worth bearing in mind that *g_s_* is fundamentally determined by stomatal behaviour and pore width and compensatory mechanism between density and behaviour can exist. For example work by [48] showed that reducing SD (by overexpressing the STOMATAL DENSITY AND DISTRIBUTION (SDD1) gene) in Arabidopsis, did not reduce *g_s_* as expected, because an increase in stomatal aperture compensated for the lower SD and, therefore, there was no difference in *g_s_* between the mutants and controls.

Overcoming the stomatal aperture/stomatal density trade-off was successfully shown by [49], whereby downregulation of either the α-subunit or β-subunit of farnesyltransferase (ERA1) increased stomatal sensitivity to ABA in canola. The increased ABA sensitivity reduced *g_s_*, and facilitated yield maintenance in plants subjected to drought conditions through improved resource use. Increased *g_s_* has been achieved through a number of metabolic manipulations, for example, silencing a mitogen-activated protein kinase MPK4 in *Nicotiana attenuata* increased *g_s_* and *A* threefold, as well as increased sensitivity to water stress [50]. In rice [51], tomato [52] and grapevine [53] aquaporin overexpression increased *gs* and *A*, both under optimal and stress conditions. These studies clearly demonstrate the potential of manipulating stomatal characteristics to improve carbon assimilation and resource use. However, restrictions on growing GM crops in many countries (particular in Europe) mean that alternative methods for manipulating *g_s_* need to be realised. This could be achieved by exploiting the significant natural variation in stomatal characteristics and behaviour that is known to exist. However, in order to achieve this, a greater understanding of the underlying genetics that control variation as well as the compensatory mechanisms between stomatal anatomy and behaviour need to be fully understood.

## Natural variation in *g_s_* and genetic control for selection

Large natural variation in *g_s_* under optimal, steady-state light conditions has been shown for a range of crops. In Table 1, some of the most significant and recently reported work on the variation in *g_s_* is summarized.

**Table 1:** Examples of variation assessed and the range of *g_s_* detected in cultivars or populations of different crops. The experimental design and methods for *g_s_* estimation are shown

Authors	Crop	*g_s_* range (mol m^−2^ s^−1^)	Experimental material and analysis
[54]	Wheat	0.15–0.55	Chromosome substitution lines grown under field conditions with and without supplementary irrigation. *g_s_* analysed with Li-Cor 6400 at saturating light
[55]	Wheat	0.10–0.42	Field experiment. Double haploid population grown under supplementary irrigation and no irrigation treatment. *g_s_* estimated with CI-340 portable gas-exchange system at saturating light
[7]	Spring wheat	0.34–0.57	Historical selection of wheat cultivars grown over three field seasons. *g_s_* analysed with steady state porometry on both adaxial and abaxial surface
[56]	Durum wheat	0.25–0.42	Historical selection of Italian cultivars grown over two growing seasons. *g_s_* estimated with CIRAS-1 under natural light conditions
[16]	Rice	0.25–0.85	64 accessions from a rice diversity research set of germplasm and 3 high-yielding cultivars grown under field conditions. *g_s_* estimated with Li-Cor 6400 at saturating light
[63]	Rice	0.12–0.21	Field screening under optimal and water stress condition of a BC_3_F_6_ mapping population. *g_s_* analysed with Li-Cor 6400 at near-saturating light
[62]	Soybean	0.40–0.65	Greenhouse experiments including VPD manipulation and water stress application on eleven cultivars. *g_s_* analysed with Li-Cor 6400 at saturating light
[65]	Cotton	0.51–0.82	Field grown segregating population. *g_s_* analysed with steady-state porometer
[57]	Cotton	0.70–0.85	Field grown historical selection of cotton. *g_s_* estimated during sunny days with Li-Cor 1600 steady state porometry
[67]	Cotton	0.25–0.75	Field experiment on obverse and reverse F_1_ lines. *g_s_* analysed with Li-Cor 6400 diurnally and at different light intensities and temperatures.
[58]	Tomato	0.80–1.20	Historical selection of tomatoes cultivars grown in the field and the greenhouse. *g_s_* was analysed in the field with a Li-cor 6400 at saturating light

Potentially useful genomic regions have been identified that could provide crucial information for future breeding programmes. For example in cereals, variation in radiation use-efficiency [59], canopy temperature and yield [7] have been attributed to differences in *g_s_*, signifying the importance of this trait for possible further yield progress. Indeed, [7] showed that the year of release and yield genetic gain in wheat were linearly related with *g_s_* thus illustrating that the increase in yield was achieved by inadvertently selecting for high *g_s_*, cooler canopy and inevitably higher *A*. A large normally distributed phenotypic variation for *g_s_* was reported in two segregating populations of wheat, illustrating potential quantitative inheritance and a heritability on a family mean basis of up to 73% [60]. Subsequently, it has been shown that *g_s_* is subject to a polygenic control which was in turn associated with QTLs for yield under stress conditions [61]. Therefore, there is strong evidence that variation in *g_s_* is present in wheat and that marker-assisted selection could be carried out if more accurate genomic regions controlling *g_s_* are detected.

Genotypic differences in *g_s_* have also been detected in eleven soybean genotypes analysed under saturating light with different soil water conditions, which lead to variation in *W_i_* in response to water stress [62]. Anatomy-driven variation in *g_s_* was shown to be present in elite rice cultivars [16], and QTLs for steady-state *g_s_* at saturating light in introgression lines under water stress conditions were identified on chromosomes 3 and 9 [63]. Other QTLs related to *g_s_* were identified in rice [64] and cotton [65], thus suggesting the possibilities of selection for *g_s_* through marker-assisted selection in several crops. Other sources of potential variation in *g_s_* (and thus *A*) include inter-specific and inter-generic crosses within the *Triticeae* [66]. The use of F_1_ hybrids in crops where heterosis for *g_s_* is present (e.g. cotton; [67]) has also been shown to be successful. Hence, variation in *g_s_* is already present in many crops with potential to be included in breeding programmes for both yield potential and enhancement in stress tolerance. Moreover, although previous research has put a great deal of emphasis on assessing the variation in stomatal anatomical characteristics or steady-state *g_s_*, there is limited information regarding potential intra-specific variation in the rapidity of stomata responses in major food crops, with some information available in rice only [39^••^]. Further work needs to focus on detecting the genetic basis of stomatal rapidity, thus enhancing the ability for selection of more efficient crops under naturally dynamic environmental conditions.

## Conclusions

Stomatal conductance is a major determinant of photosynthesis, and there is clear evidence that manipulating *g_s_* can improve crop performance and yield. Natural variation in *g_s_* exists in crops, with several genomic regions identified that could provide unexploited targets for ongoing breeding programmes. Additionally the rapidity and kinetics of stomatal responses to changing environmental conditions have been demonstrated to greatly impact *A* and water use, and are the result of differences in anatomical and biochemical stomatal components [9^••^]. As higher stomatal density is often correlated with smaller stomata, and smaller stomata have been reported to respond more rapidly to changing environmental cues, a future priority could be the selection of cultivars with these anatomical features or the identification of the genomic regions that correspond to such traits of interest. Guard cell biochemistry and the density and activity of membrane transporters play a key role in both the magnitude and rapidity of *g_s_* responses, representing novel targets for improving crop productivity, although little is known regarding natural intra-specific variation in these functional traits. Future breeding programmes should consider the integration of *both* density and behavioural beneficial traits so that equal consideration is given to the magnitude and rapidity of *g_s_* responses, as well as the overall steady state *g_s_* value. In conclusion intra-specific variation in the key components governing stomatal dynamics and overall *g_s_* represent an unexploited target for improving *A* and *W_i_* for increased plant productivity.

## References and recommended reading

Papers of particular interest, published within the period of review, have been highlighted as:

• of special interest
•• of outstanding interest
